# The Role of TREM2 in Alzheimer’s Disease and Other Neurological Disorders

**DOI:** 10.4172/2161-0460.1000160

**Published:** 2014-10-06

**Authors:** Faris Yaghmoor, Ahmed Noorsaeed, Samar Alsaggaf, Waleed Aljohani, Henrieta Scholtzova, Allal Boutajangout, Thomas Wisniewski

**Affiliations:** 1 Departments of Neurology, New York University School of Medicine, Alexandria ERSP, 450 East 29th Street, New York, NY 10016, USA; 2 Pathology, New York University School of Medicine, Alexandria ERSP, 450 East 29th Street, New York, NY 10016, USA; 3 Psychiatry, New York University School of Medicine, Alexandria ERSP, 450 East 29th Street, New York, NY 10016, USA; 4 Physiology and Neuroscience, New York University School of Medicine, Alexandria ERSP, 450 East 29th Street, New York, NY10016, USA; 5 King Abdulaziz University, School of Medicine, Jeddah, Saudi Arabia

**Keywords:** TREM-2, Alzheimer’s disease, Fronto-temporal dementia, Nasu-hakola disease, Hereditary diffused leukoencephalopathy with spheroids, Parkinson’s disease, Amyotrophic lateral sclerosis

## Abstract

Alzheimer’s disease (AD) is the leading cause of dementia worldwide. Late-onset AD (LOAD), is the most common form of Alzheimer’s disease, representing about >95% of cases and early-onset AD represents <5% of cases. Several risk factors have been discovered that are associated with AD, with advancing age being the most prominent. Other environmental risk factors include diabetes mellitus, level of physical activity, educational status, hypertension and head injury. The most well known genetic risk factor for LOAD is inheritance of the apolipoprotein (apo) E4 allele. Recently, rare variants of TREM2 have been reported as a significant risk factor for LOAD, comparable to inheritance of apoE4. In this review we will focus on the role(s) of TREM2 in AD as well as in other neurodegenerative disorders.

## Introduction

AD is the leading cause for dementia in the world, and affects an estimated 5.4 million Americans currently. This figure includes 5.2 million individuals aged ≥ 65 years and ~200,000 individuals under the age of 65 [1]. AD is characterized by an imbalance between the production and clearance of amyloid β (Aβ) and tau proteins, which ultimately leads to the abnormal accumulation of these proteins in the form of senile plaques and neurofibrillary tangles, where aggregated forms of Aβ and tau deposit, respectively [2]. Soluble oligomeric forms of Aβ and tau are felt to be the most toxic species linked to the neuronal dysfunction/death in AD [3]. There are two forms of AD, the early-onset (EOAD) form, which is related to mutations in presenilin 1, presenilin 2 (PS1 and PS2) or the amyloid precursor protein (APP), when associated with autosomal dominant inheritance [4–6]. EOAD affects a minority of AD patients. Epidemiological data suggests that apparent autosomal dominant transmission is found in only ~10% of all EOAD cases, leaving the genetic association of the majority of EOAD unexplained [4,7]. The other form is the sporadic late-onset form (LOAD), which afflicts >95% of patients with AD [4–6,8,9]. Some of the known environmental risk factors for LOAD include diabetes mellitus, level of physical activity, educational status, hypertension and head injury [10]. The strongest identified genetic risk factor for LOAD is the inheritance of the apolipoprotein (apo) E4 allele [11]. Recently, rare variants of the gene encoding triggering receptor expressed on myeloid cells 2 (TREM2; located on 6p21.1) have been reported as a significant risk factor for LOAD, with an odds ratio similar to apoE4 [12]. In this review we will review the general functions and characteristics of TREM2 and related genes, mainly focusing on their association as a risk factor for AD, as well as other central nervous system (CNS) diseases.

## TREM2 Overview

**Figure 1:**
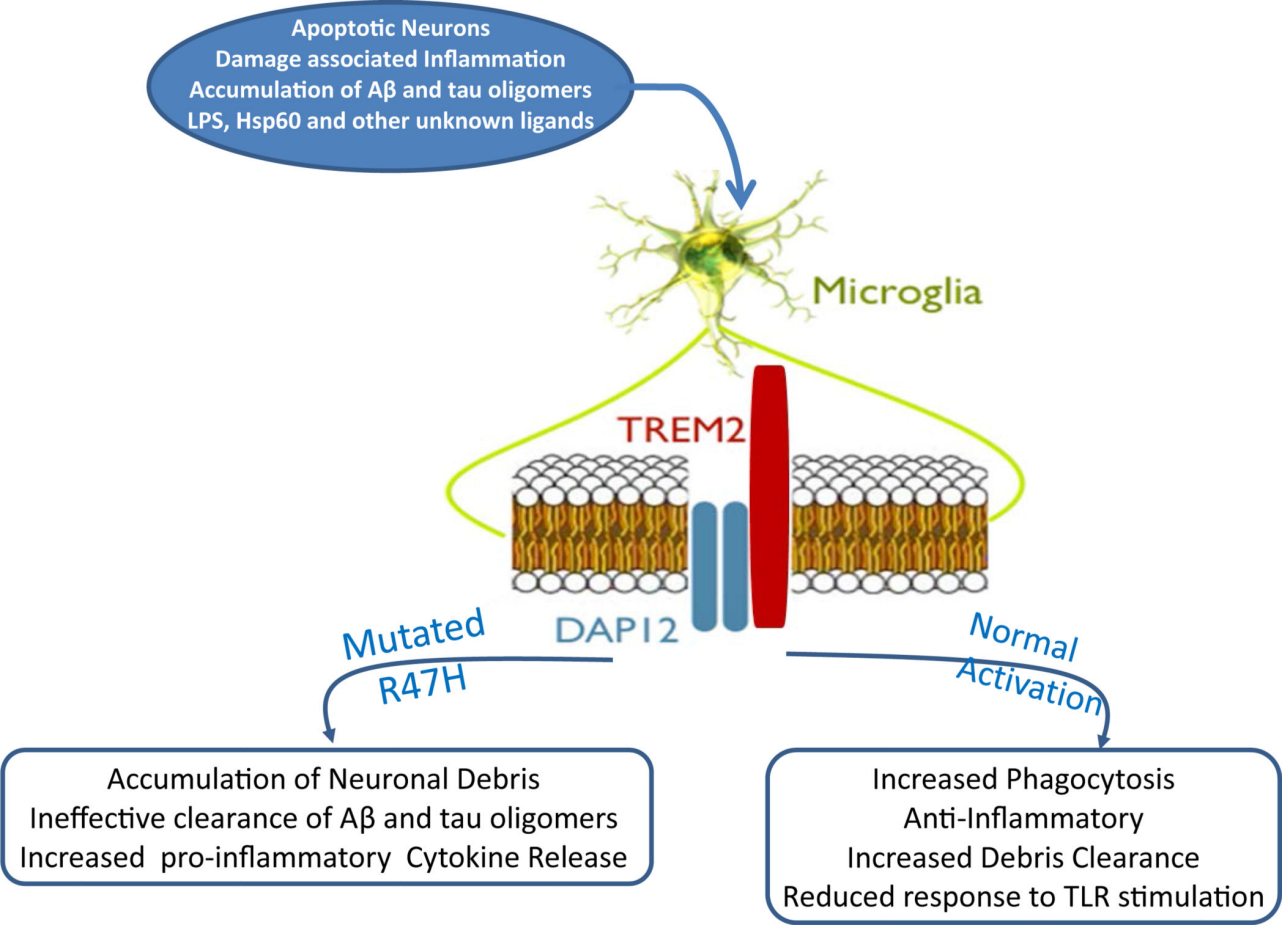
Normal and disease associated pathways of TREM2 activation.

TREM2 is an innate immune receptor expressed on the cell surface of microglia, macrophages, osteoclasts and immature dendritic cells [12–14]. TREM2 has also been found on bronchial epithelial cells, fibroblasts, and lung adenocarcinoma cells. The TREM2 gene encodes 5 exons that code for a 693 pb DNA, located on chromosome 6p21.1, which is translated into 230 amino-acids [15–17]. The receptor is a variably glycosylated, single-pass type I membrane glycoprotein made up of an extracellular immunoglobulin-like domain, a transmembrane domain and a cytoplasmic tail, which associates with tyrosine kinase-binding protein (TYROBP, also known as DAP12), forming a receptor-signaling complex[18,19] (Figure 1). TREM2 is one of the highest expressed cell surface receptors on microglia and is >300 fold enriched in microglia versus astrocytes [20]. Microglia plays a key role in the immune response in the central nervous system (CNS) and is the resident innate immune cells responsible for the early control of infections. In the human brain, TREM2 is found at high concentrations in white matter, the hippocampus and the neocortex, but at very low concentrations in the cerebellum. These regions are consistent with the distribution of pathology in AD [14,18,21]. TREM2 was initially identified as a phagocytic receptor of bacteria [22]. TREM2 recognizes anionic lipopolysaccharide (LPS) in the cell wall of bacteria. When the bacteria bind to TREM2 on macrophages, activation of the signaling pathway triggers the phagocytic uptake of the bacteria and the release of reactive oxygen species [23]. Heat shock protein 60 (Hsp60) is a mitochondrial chaperone that has also been shown to be a TREM2 agonist when expressed on the surface of neuroblastoma cells or astrocytes [24]. The formation of amyloid plaques in an AD model has been shown to induce expression of TREM2, in particular among microglia in the outer zone of plaques, correlating with partial amyloid phagocytosis [25]. TREM2 expression also correlated positively with microglia being able to stimulate CD4+ T-cell proliferation, tumor necrosis factor, but not interferon γ; hence, potentially promoting neuroprotective “wound repair responses” [25]. TREM2 has also been shown to be involved in phagocytosis of apoptotic neurons, since down regulation of TREM2 or DAP12 in microglia reduces such phagocytosis, while over expression of TREM2 has the opposite effect [26]. Other pattern recognition receptors which have been shown to play an important part in macrophage/microglial function and have a role in AD related pathology are the Toll-like receptors (TLRs) [12,27–29]. TLRs interact with the TREM2/DAP12 on multiple levels; these interactions appear to be tissue and receptor specific [30,31].

In addition to its role as part of the innate immune systems response to pathogens, TREM2 is known to have anti-inflammatory properties; it suppresses inflammatory responses by repression of cytokine production and secretion [32]. TREM2 reduces macrophage activation and inhibits cytokine production in response to both TLR2 and TLR4 ligands zymosan and LPS [33,34]. Conversely reduction of TREM2 expression by either RNA interference or by targeted gene deletion amplified inflammatory cytokine responses by macrophages following stimulation of multiple different TLRs including TLR2, 4 and 9 [35]. Modulation of innate immunity via TLR2,4 and 9 signaling pathways has previously been shown to be critical in modulating Aβ deposition. TLR4 deficient mice displayed increases of diffuse Aβ and fibrillar Aβ deposits compared with control mice [36], suggesting that TLR4 signaling is involved in Aβ clearance [37]. Microglia deficient in TLR2, TLR4, or the co-receptor CD14 are not activated by Aβ and do not show a phagocytic response [38]. Transgenic AD mice lacking TLR4 have markedly elevated levels of diffuse and fibrillar Aβ. Furthermore, stimulation of microglial cells with TLR2-, TLR4-, or TLR9- specific agonists accelerates Aβ clearance both in vitro and in vivo [39]. We have shown that the administration of the TLR9 agonist CpG oligonucleotides (ODN) containing unmethylated CpG sequences to AD model Tg2576 mice induced a reduction of cortical and vascular Aβ levels without apparent toxicity and improve cognitive function [40]. In addition, TLRs can affect tau related pathology; TLR4 ligand (LPS)-induced MAPT hyperphosphorylation and exacerbation of tau pathology has been well documented [41–44]. Hence it can be speculated that TREM2 has a protective role in AD pathogenesis. Its anti-inflammatory properties could reduce innocent bystander neuronal damage, as well as, having a role in modulating TLR related signaling pathways that affect both Aβ and tau deposition [12,16,17,23,25]. TREM2 is also known to effect phagocytosis of damaged cells. TREM2 interacts with endogenous ligands on neurons, leading to the direct removal of damaged cells [45]. In several models of multiple sclerosis increased microglial expression of TREM2 has been shown to enhance phagocytosis and promotes a M2-like activation state of microglia, which is thought to have protective effects [46–48]. The removal of damaged or apoptotic neurons mediated via TREM2 could promote tissue repair in response to AD related pathology. This TREM2 mediated phagocytic activity also has been linked to an enhanced ability of microglia to clear Aβ and amyloid plaques in vitro and in AD model APP23 Tg mice [25]. The importance of TREM2 is not confined to the innate immune response to Aβ pathology. A recent large GWAS study has shown that the TREM2 R47H variant has a strong association with both elevated CSF tau and hyperphosphorylated tau protein (ptau) levels [49]. This is important as numerous studies have shown that increases of ptau in CSF correlates with neuronal loss and is predictive of cognitive decline in AD [50–52]. Furthermore, neurofibrillary tangle deposition correlates better with the degree of dementia, compared to the amyloid plaque burden [2]. Microglia are well known to have the potential to acquire a broad array of cytotoxic and cytoprotective functional states [41,48,53]. TREM2 appears to be an important factor regulating this balance in response to AD associated pathology.

## TREM2 and the Risk for AD

Jonsson et al. performed whole genome sequencing on 2261 Icelandic individuals and found that a rare mutation (rs75932628-T, frequency of 0.63%), predicted to result in a TREM2 R47H substitution, was associated with an increased risk of AD (odds ratio 2.92). Subsequently this association was replicated in cohorts from the USA, Germany, the Netherlands and Norway [18]. Concurrently, Guerreiro et al. confirmed the link between LOAD and the R47H variant by meta-analysis of three imputed data sets of genome-wide association studies (EADI, GERAD and ANM) [19]. They also found six additional variants (Q33X, Y38C, T66M, D87D, R98W and H157Y) that were present in affected cases and not in controls, which could be related to AD pathology. Three of these variants (Q33X, Y38C and T66M) had been previously reported in the homozygous state to be associated with a frontotemporal dementia like syndrome [54]. A replication study conducted in a Spanish population confirmed the variant to be associated with a higher risk for LOAD, as well as, EOAD, with R47H found exclusively in 1.4% of AD cases.

T66M, Y38C, and Q33X homozygous variants have also previously been observed in Nasu-Hakola Disease (NHD) and are strongly suspected to result in TREM2 loss-of- function [19].

The R47H variant has a minor allele frequency (MAF) of 0.63% in Icelanders, 0.26% in European Americans, and 0.2% in African Americans[18]. Another research group in France confirmed the association between AD with the TREM2 R47H substitution variant (rs75932628-T), located within the extracellular immunoglobulin-like domain-mutation [55]. This study’s sample included 726 EOAD and 783 controls. The effect size was similar to the reports discussed above for TREM2 in LOAD, suggesting that the role of TREM2 on AD pathology is not critically dependent on aging. Molecular dynamics simulations have suggested that the R27H substitution could have significant effects on ligand binding affinity, as well as the structural configuration of TREM2 [56]. Assuming that the TREM2 risk variant impairs TREM2 function, it is strongly believed that it does so by decreasing affinity of TREM2 to its natural ligands. This will in turn decrease its downstream effect. This suggests that reduced function of TREM-2 causes reduced phagocytic clearance of amyloid proteins or cellular debris. This then impairs the protective mechanism in the brain, ultimately leading to the abnormal accumulation of tau and beta-amyloid, the hallmark of AD.

A recent study has also shown that TREM2 undergoes sequential proteolytic processing by ectodomain shedding and intramembrane proteolysis [57]. The latter cleavage is by γ-secretase, which is the enzyme that also cleaves the amyloid precursor protein to release Aβ [58]. Inhibition of γ-secretase produces an accumulation of TREM2 carboxyl terminal fragments at the cell surface, trapping its adaptor DAP12, and resulting in impairment of TREM2 signaling [57]. The γ-secretase complex contains either PS1 or PS2 as the catalytically active component, with mutations in PS1/2 being the most common cause of EOAD [6, 59]. These findings provide another link between TREM2 and pathways involved in AD pathology.

## The TREM Family and a Missense Variant Protective against Alzheimer’s Disease

TREM2 belongs to a family of structural related genes clustered on human chromosome 6p21.1 and mouse chromosome 17C. These include TREM1, TREM2, and TREM3, as well as ‘TREM-like” genes: TREML1 and TREML2 [60–62]. The first to be discovered was TREM1 which has been established as an amplifier of systemic inflammatory responses [15,62]. This contrasts with the role of TREM2 which has emerged as a negative regulator of autoimmunity [61,62]. An AD association GWAS had identified an inter-genic SNP [rs9381040] located 5.5 Kb downstream from TREML2 and 24 Kb upstream from TREM2 [63]. A recent exome-sequencing study of 16,254 cases and 20,052 controls suggest a TREML2 coding missense variant p.S144G as the driver of the latter GWAS signal, which is independent of the TREM2 R47H loci [64]. This variant is associated with a reduced risk for AD [OR=0.91; CI=0.86–0.97] [64]. TREM2 and TREML2 appear to have opposing roles in their modulation of innate immunity. Treatment of microglia with IL-1β represses expression of TREM2, while increasing expressing of TREML2 [64]. Unlike TREM2, TREML2 signal is not coupled to DAP12 and it appears to play a pro-inflammatory role [61,64]. Hence different missense variants in the TREM family can either enhance AD pathology or inhibit it; highlighting the importance of innate immunity modulation in the pathogenesis of AD.

## TREM 2 and Other CNS Diseases

### Nasu-Hakola Disease: Polycystic lipomembranous osteodysplasia with sclerosing leukoencephalopathy

Homozygous TREM-2 mutations that cause a near-complete functional loss of the TREM-2 gene (e.g. p.Q33X) or TYROBP/DAP12 have been known to be linked to an autosomal recessive disorder called polycystic lipomembranous osteodysplasia with sclerosing leukoencephalopathy (PLOSL), also known as Nasu-Hakola Disease [65–67]. Over 200 cases have been reported worldwide in the literature, the majority of them being in the Japanese and Finnish population. The prevalence in Finland is estimated between 1/500,000 and 1/1,000,000. This fatal disease is characterized by manifestations affecting both bones and brain suggesting that the function of TREM-2 is similar in both systems. Patients with (PLOSL) have progressive presenile inflammatory neurodegeneration that leads to dementia and formation of multifocal bone cysts predisposing to pathological fracture. In addition, these patients often have psychiatric symptoms in the second decade of life, followed by severe frontotemporal dementia with premature death in the fourth or fifth decade of life. PLOSL patients have not been reported to have AD amyloid plaques, indicating that dysfunctional neuroinflammation can be an amyloid independent pathway leading to dementia [23]. Patients with heterozygous loss-of-function mutations carry a higher risk for age associated cognitive loss and/or LOAD [19,54,66,68]. Three TREM2 variants previously linked in the homozygous state to either PLOSL or FTD (e.g. p.T66M, p.Y38C, and p.Q33X) have been shown to be associated with LOAD. Conversely, the R47H mutation has also been reported in patients with PLOSL suggesting similar neuro-inflammatory mechanisms may mediate neuronal dysfunction/death in AD and PLOSL, in association with amyloid deposition or in its absence, respectively [19].

## Hereditary Diffuse Leukoencephalopathy with Spheroids

The colony-stimulating factor 1 receptor (CSF1R) is located on microglia. It binds CSF1 and like TREM2 co-signals through DAP12. Patients with a partial loss-of-function of this gene develop a neurodegenerative disease called hereditary diffuse leukoencephalopathy with spheroids (HDLS) [69]. This autosomal dominant disease is characterized by the degeneration of white matter predominantly in the frontal lobes and corpus callosum, with subsequent cortical atrophy [70,71]. The clinical manifestations are variable and can include behavioral changes, dementia, depression, parkinsonism, ataxia, pyramidal signs, and seizures [70,71]. Neuropathologically there is widespread myelin and axonal destruction, in association with axonal swellings called spheroids, as well as characteristic pigmented macrophages [70,71]. The swelling in the axons resemble, to some extent, those produced by shear stress in closed head injuries with damaged axons. A number of different loss of function mutations in CSF1R have been reported, with in vitro studies suggesting that patients with ≤50% of the normal protein will manifest the disease [72]. It has been speculated that impaired CSF1R mediated microglial repair of axonal degeneration is the mechanism underlying HDLS [71,72]. Hence dysfunction of the innate immune complex consisting of TREM2, CSF1R and the signaling molecule DAP12 in microglia can lead to chronic neurodegeneration, with variable clinical and pathological phenotypes.

## Parkinson’s disease, Frontotemporal Dementia and Amyotrophic Lateral Sclerosis

Parkinson’s disease (PD), frontotemporal dementia (FTD) and amyotrophic lateral sclerosis (ALS), similar to AD, all belong to the category of conformational neurodegenerative disorders where normal self-proteins aggregate forming toxic β-sheet rich intracellular inclusions or extracellular amyloid deposits [3,74]. The most toxic species of the different aggregates are felt to be oligomers, which in some cases can spread using a prion like mechanism [3, 75]. In PD α-synuclein aggregates forming oligomers and Lewy bodies, while in different forms of FTD tau, TAR-DNA-binding protein-43 (TDP-43) or fused in sarcoma (FUS) can aggregate[73,76]. In different forms of ALS, SOD1, TDP-43 or FUS form toxic aggregates [77]. Microgliosis is a critical part of each of these disorders and the activation state of microglia can result in either pathology enhancement or amelioration [53]. TREM2 variants have recently been associated with each of these disorders. In PD the R47H TREM2 variant was first found to be associated when patient populations were screened in the USA and Spain [78]. A subsequent study has confirmed the association of the R47H variant to PD with an odd ratio of 2.67 [79]. TREM2 variants are also associated with a FTD phenotype. Besides the association of TREM2 variants with PLOSL, a homozygous deletion of the consensus donor splice site in intron 1 of TREM2 was reported in a Lebanese family with typical behavioral FTD with no bone involvement [80]. A typical autosomal recessive FTD phenotype has also been associated with a p.Y198X TREM2 mutation in a Columbian family [81]. Additional studies have reported an association with a FTD phenotype and the R47H TREM2 variant, with the frequency of the variant being ~ three fold over represented among FTD patients, with an odds ratio of 5.06 [79,82]. A recent study has also shown a link between the R47H TREM2 variant and ALS [83].

## Conclusions

Studies conducted over 20 years ago had suggested the potential critical role of microglia for both the formation and clearance of amyloid lesions in AD(84–86). Interest in the importance of innate immunity modulating neurodegeneration has been greatly increased by recent GWAS studies that have linked genes such as CR1, CD33 and MS4A4A/MS4A6A that are associated with microglial function to AD [87,88]. Numerous studies on the relationship of TLRs to AD have shown that modification of these signaling pathways can have profound effects on AD related pathology, through modification of the inflammatory state of microglia/macrophages. Our own studies have shown that appropriate stimulation of innate immunity via TLR9 can ameliorate both Aβ and tau related pathology [12,40,89]. Recent studies, outlined above, have linked TREM2 variants to most of the different types of conformational neurodegenerative disorders. These studies indicate that modification of microglial function in neurodegeneration is a critical therapeutic target.
